# Constuction Notes

**Published:** 1894-11-10

**Authors:** 


### CONSTRUCTION NOTES.

**New Wing to Victoria Hospital, Swindon.**

So much have the claims upon this hospital grown that the need of an increased area has for some time been felt. Not only has the accommodation for patients proved insufficient, but the fact of the operating-room being upstairs instead of on the ground floor was an additional argument in favour of completing the original design of the hospital by the construction of the new wing. Working from plans supplied by Mr. W. H. Read, architect, Mr. T. Barrett, who secured the contract, has erected most of the shell of the new wing. This extension will resemble, outwardly, the existing building, and will contain two wards of six beds each. One ward only will be furnished for use on completion; increased bed-room accommodation for staff has also been provided for. There will also be several alterations in the present building which could not be carried out before, owing to want of space. The nurses' present sitting-room is also being enlarged.

**New Infectious Hospital at Old Swan.**

This new hospital has been built for infectious diseases at Mill Lane, Old Swan, at the instance of the members of the West Derby and Wavertree Joint Hospital Committee. The various blocks of buildings, six in all, forming the institution are erected on a site at the corner of Mill Lane and Burns Road, with entrance from the corner, and consist of a porter's lodge and the administration block, bath and dressing block, laundry, one pavilion for eight beds and two pavilions for twelve beds each, making thirty-two beds in all for this, the first part of the scheme. The buildings have been so arranged on the site as to leave room for the erection of three more pavilions, two for twelve beds and one for ten beds, making the accommodation of the complete hospital sixty-six beds. The whole site is enclosed by a wall seven feet high. The administration block is cellared throughout, and on the ground floor is the matron's sitting-room, overlooking the principal entrance, waiting-room for visitors, doctor's-room, dispensary, and a committee-room. The kitchen is also on this floor, a well lighted apartment, with scullery, larder stores, &c. Next to the kitchen is a service corridor, bed-rooms for the matron and general staff, with linen store, bath-room, housemaids' sink, &c., occupying the whole of the first floor. The kitchen portion of the building is made large enough to work the complete scheme, and whenever the hospital pavilions are completed, the extra accommodation required for the staff will be provided in a wing built at the rear and down the Burns Road side. There is a small isolated building on the south side and near the entrance, containing a bath-room and two dressing-rooms, where patients are required to have a bath before leaving the hospital. The pavilions are so placed on the site that the windows of the wards face somewhat to the S.E., securing the largest possible amount of sunlight. The pavilion nearest to the entrance is intended for the reception of doubtful cases, and contains two single bed wards, and one two bed ward for men with all necessary offices. A lavatory and sink, &c., are provided at the end of each ward, approached across a ventilated corridor. Both these pavilions are carried upon open arches. The laundry block consists of a perfectly-equipped washhouse, fitted up with airing stoves, &c., by Messrs. Braby and Co. There is also a mortuary with the necessary appliances for post-mortem examinations. An ambulance shed is also provided. At the further end of the building is the boiler and steam-disinfecting plant of the most approved order, supplied by Messrs. Manlove and Co. The sole contract for this hospital was taken by Mr. Samuel Webster, of Bootle, from plans and under the direct supervision of the architects, Messrs. C. O. Ellison and Son, 22, Sir Thomas Street, Liverpool.

